# Assessment of Preprocedural Factors Associated with 5-Year Complete Response After Transarterial Radioembolization in Patients with Hepatocellular Carcinoma

**DOI:** 10.3390/diagnostics15182297

**Published:** 2025-09-10

**Authors:** June Park, Dong Kyu Kim, Seungsoo Lee, Shin Hye Hwang

**Affiliations:** 1 Department of Radiology, Severance Hospital, Research Institute of Radiological Science, Yonsei University College of Medicine, Seoul 03722, Republic of Korea; radiopark1984@yuhs.ac; 2 Department of Radiology, Yongin Severance Hospital, Research Institute of Radiological Science, Yonsei University College of Medicine, Yongin 16995, Republic of Korea; olclocr@yuhs.ac (S.L.); pleiades5@yuhs.ac (S.H.H.)

**Keywords:** hepatocellular carcinoma, transarterial radioembolization, tumor response, tumor diameter, portal vein thrombosis

## Abstract

**Background**: There is little evidence available regarding the long-term tumor response after transarterial radioembolization (TARE) for hepatocellular carcinoma (HCC). **Aim**: To identify preprocedural predictive factors for achieving a 5-year complete response (CR) following TARE in patients with HCC. **Methods**: This retrospective study included 37 patients with treatment-naïve HCC who underwent TARE between January 2016 and December 2019 and were followed for at least 5 years. Tumor characteristics—including maximum diameter, number of main lesions, presence of satellite nodules, and portal vein thrombosis—were evaluated using preprocedural liver dynamic magnetic resonance imaging. Treatment response was assessed according to the modified Response Evaluation Criteria in Solid Tumors. Multivariate logistic regression analyses were performed to identify factors associated with tumor response following TARE. **Results**: Thirty-seven patients (median age, 64 years) were categorized into two groups: (1) the CR group (n = 9), consisting of patients without tumor recurrence for 5 years and without additional treatment; and (2) the non-CR group (n = 28), consisting of patients who required additional treatment because of residual or recurrent viable tumors. Tumors in the non-CR group had significantly larger diameters compared with those in the CR group (9.8 cm vs. 5.9 cm, *p* = 0.006). In multivariable analysis, a tumor diameter > 7 cm was the only factor significantly associated with tumor recurrence (odds ratio = 21.277, *p* = 0.010). Portal vein thrombosis did not reach statistical significance (odds ratio = 9.779, *p* = 0.063). **Conclusions**: Tumor diameter > 7 cm is a significant predictor of tumor recurrence within 5 years after TARE for HCC. This finding may support a more individualized post-TARE management approach, potentially allowing clinicians to avoid overtreatment and adopt a watchful waiting strategy for selected patients.

## 1. Introduction

Hepatocellular carcinoma (HCC) is the most common primary liver malignancy and third leading cause of cancer-related mortality worldwide [1]. While curative treatments, such as surgical resection and liver transplantation, offer the best survival outcomes, most patients with intermediate-stage unresectable HCC require locoregional therapies, such as transarterial chemoembolization (TACE) or transarterial radioembolization (TARE) [2,3]. Although TACE is a well-established treatment for intermediate-stage HCC, TARE using yttrium-90 is increasingly employed for tumor downstaging and as a bridge to liver transplantation in patients with intermediate-stage disease, as well as in those with portal vein thrombosis at the advanced stage [4,5]. Furthermore, TARE has gained attention as a promising modality for managing early-stage disease [6,7].

Following HCC treatment, response assessment is most commonly performed using either the modified Response Evaluation Criteria in Solid Tumors or Liver Imaging Reporting and Data System Treatment Response Algorithm [8,9]. However, accurate evaluation of early tumor response after TARE is challenging because of persistent peritumoral hemorrhage, edema, and equivocal hyperenhancement caused by radiation effects, which can persist for up to 3 months on follow-up computed tomography or magnetic resonance imaging (MRI) [10,11]. Therefore, in clinical practice, if a definite complete response (CR) is not observed on imaging and tumor markers fail to normalize within 3–6 months after TARE, clinicians typically proceed with additional interventions, such as TACE or surgical resection. Consequently, most published studies emphasize short-term tumor response after TARE, whereas investigations of long-term outcomes remain relatively limited [12,13,14]. However, most existing long-term follow-up studies have focused on overall survival, with findings limited to indicating that overall survival after TARE is comparable to resection or TACE [15,16], without identifying preprocedural tumor factors with a significant association with overall survival [17]. There was not much published information about long-term treatment response or preprocedural predictors of achieving a 5-year CR after TARE in patients with HCC. Assessing the 5-year tumor recurrence after treatment is widely regarded as clinically meaningful, since for many cancers, including HCC, the absence of recurrence within this timeframe strongly suggests a low likelihood of late recurrence, even though some cases may still recur beyond five years [18,19]. 

Traditional treatment models for HCC have not fully reflected the heterogeneity of patients or predicted individual variability in treatment response, which has limited progress toward personalized therapeutic strategies [20,21]. In the context of TARE, identifying factors that can predict a CR over a 5-year period without the need for additional treatment may help avoid overtreatment. Moreover, these predictive factors can play a pivotal role in facilitating individualized treatment planning and improving the accuracy of patient selection and prognostication.

Therefore, the aim of this study was to identify preprocedural factors associated with a 5-year CR to TARE in patients with HCC.

## 2. Subjects and Methods

### 2.1. Study Patients

This single-center retrospective study was approved by the Institutional Review Board of Severance Hospital, and the requirement for informed consent was waived (No. 4-2024-0810). This study was performed in accordance with the ethical principles of the Declaration of Helsinki and Good Clinical Practice guidelines.

**Figure 1:**
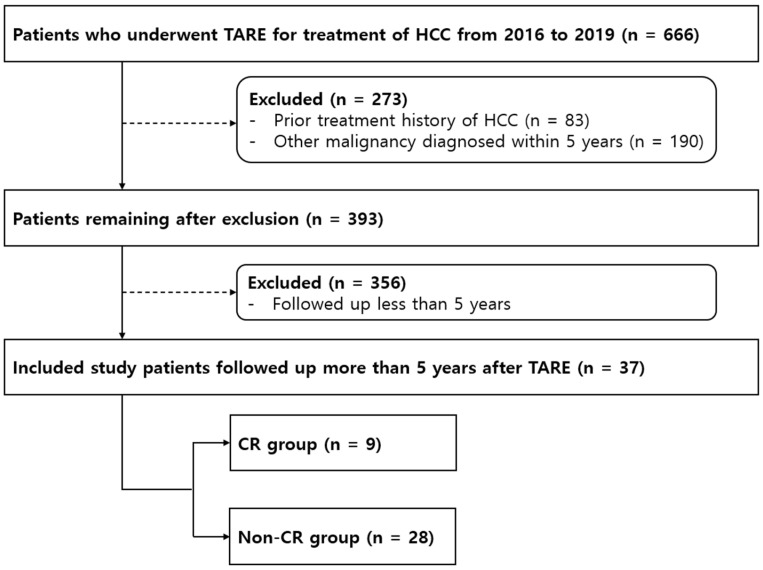
Flow chart of the study population. TARE, transarterial radioembolization; HCC, hepatocellular carcinoma; CR, complete response.

The database of consecutive patients who underwent radioembolization in a single tertiary center from January 2016 to December 2019 was reviewed. After excluding the patients with cholangiocarcinoma or metastatic liver cancer. there were 666 patients who underwent TARE. Then, those with a prior treatment history for HCC or other malignancies diagnosed within 5 years were excluded (n = 273). Among the 393 patients who remained after exclusion, 37 patients who were followed up for at least 5 years after TARE were included as the study population in this study. The study population was then classified into two groups based on modified Response Evaluation Criteria in Solid Tumors [8]: (1) CR group (n = 9), patients without tumor recurrence for 5 years without any additional treatment; or (2) non-CR group (n = 28), patients undergoing additional treatment because of remaining or recurrent viable tumors (Figure 1). HCC was diagnosed based on the typical imaging findings of liver dynamic MRI according to the Liver Imaging-Reporting and Data System [22].

### 2.2. Pre-Procedure MR Image Acquisition and Analysis

MRI examinations were performed using a 3.0-Tesla system (SIGNA Premier, GE Healthcare, Milwaukee, WI, USA; Ingenia Elition X, Philips Healthcare, Best, The Netherlands). The scan protocol included dual-echo T1-weighted gradient-echo images (in- and opposed-phases), T1-weighted three-dimensional gradient-echo images with dynamic contrast enhancement, navigator-triggered single- or multi-shot T2-weighted turbo spin echo images, diffusion-weighted images with b-values of 0, 50, and 800 mm^2^/s, and calculated ADC maps. Dynamic T1-weighted imaging was performed before and after the administration of a hepatobiliary contrast agent (gadoxetate disodium, Primovist^®^; Bayer Pharma AG, Berlin, Germany). Arterial phase scanning was initiated using the test bolus or bolus tracking technique, and portal phase (60 s), 3 min delayed phase (transitional phase), and 20 min hepatobiliary phase images were evaluated. Detailed parameters of the MRI sequences are listed in Supplementary Table S1.

MR images were retrospectively reviewed independently by two experienced radiologists with 14 and 10 years of radiological experience. The reviewers knew that the patients had undergone TARE for HCC; however, they were unaware of any other clinical information (i.e., tumor size and stage). The following information was obtained from the initial imaging examinations: tumor epicenter (right vs. left), largest tumor diameter, number of main tumors (distinct HCC lesions excluding satellite nodules on baseline MRI), presence of satellite nodules (lesions ≤ 2 cm in size with similar MRI features, located ≤2 cm from the main tumor [23]), tumor enhancement pattern (typical [non-rim arterial hyperenhancement with washout on the portal or delayed phase] vs. atypical), tumor margin (smooth vs. irregular), presence of portal vein thrombosis, hepatic vein invasion of the tumor, and the amount of ascites. A consensus review was performed in case of any disagreements.

### 2.3. Transarterial Radioembolization Procedure

TARE was performed by two interventional radiologists with 13 and 25 years of experience in interventional oncology. Planning angiography and cone beam CT were performed to determine the tumor-feeding arteries through which to deliver the microspheres. Planar scintigraphy and single-photon emission CT were performed for adequate dose calculation. Both resin and glass microspheres were used mainly based on their availability during the treatment periods. Dose calculations were based on the partition model for resin microspheres and the Medical Internal Radiation Dose for glass microspheres, as recommended by the manufacturers. The microspheres were administered as selectively as possible to preserve unaffected liver tissue [24].

### 2.4. Clinical Data Collection

Clinical and laboratory data, including age, sex, model for end-stage liver disease (MELD) score, and tumor markers (serum alpha-fetoprotein and prothrombin induced by vitamin K absence or antagonist-II), were obtained from the patients’ electronic medical charts. The interval periods from the date of the pre-procedure MR examination to the date of the TARE procedure were also obtained from each patient.

### 2.5. Statistical Analysis

Continuous variables are expressed as medians with interquartile ranges, and categorical variables are expressed as absolute numbers with percentages. Continuous variables were compared using the Mann–Whitney U-test or Kruskal–Wallis test, whereas categorical variables were compared using the chi-squared test or Fisher’s exact test. Univariable and multivariable logistic regression analyses using stepwise selection of variables were performed to identify the factors associated with tumor recurrence after TARE. Factors related to tumor recurrence (*p* < 0.1) in the univariable analysis were included in the multivariable analysis. Outcomes were expressed as odds ratios (ORs) with 95% confidence intervals (CIs). All statistical analyses were performed using R version 4.3.3 software (R Foundation for Statistical Computing, Vienna, Austria), and *p* < 0.05 was regarded as statistically significant.

## 3. Results

### 3.1. Study Patients’ Characteristics

Of the 37 study patients, there was no recurrence of HCC in nine patients without additional treatment for 5 years after TARE (CR group), whereas 28 patients underwent additional treatment for recurrent HCC within 5 years after TARE (non-CR group). The diameter of the largest tumor was significantly larger in the non-CR group than in the CR group (9.8 cm [6.8–12.0 cm] vs. 5.9 cm [4.8–6.8 cm], *p* = 0.006). There were no significant differences in other clinical, radiological, or laboratory data between the two groups. Portal vein thrombosis was more frequently observed in the non-CR group than in the CR group; however, the difference was not statistically significant (46.4% [13 of 28 patients] vs. 11.1% [one of nine patients], *p* = 0.062). The baseline patient characteristics are summarized in Table 1.

### 3.2. Assessment of Predictors for Tumor Recurrence After TARE

**Figure 2:**
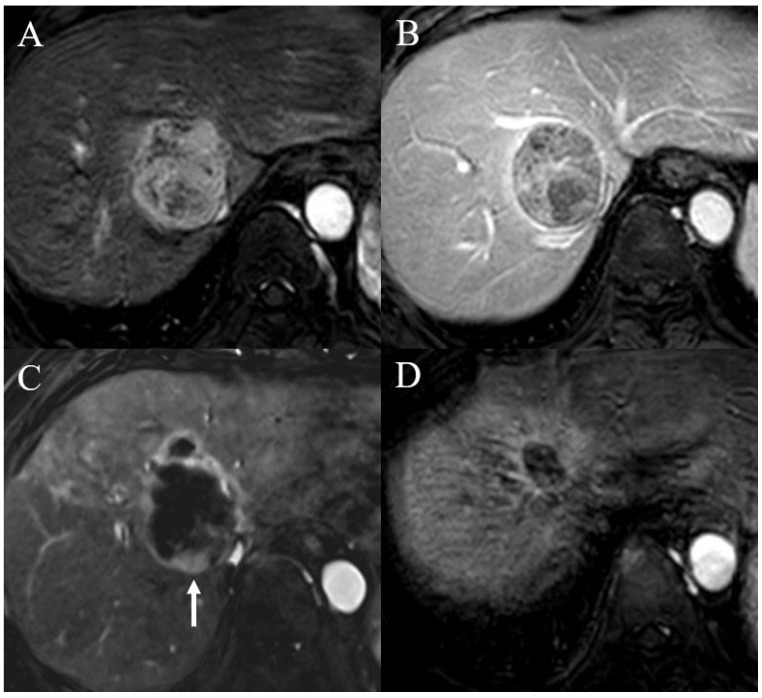
Case of a man who underwent transarterial radioembolization (TARE) for hepatocellular carcinoma. (**A**,**B**) Pre-procedure liver dynamic magnetic resonance imaging revealed a 5.6 cm hepatocellular carcinoma in segment VII. (**C**) On follow-up liver dynamic magnetic resonance imaging at 3 months after TARE, equivocal arterial enhancement (arrow) was noted at the inferior aspect of the treated lesion. The patient was followed up without any additional treatment at the discretion of the referring physician. (**D**) At 1 year after TARE, the treated lesion was markedly decreased in size, with radiation-related change in the adjacent hepatic parenchyma. The patient was followed up until recently and has remained free of recurrence for 66 months after TARE.

**Figure 3:**
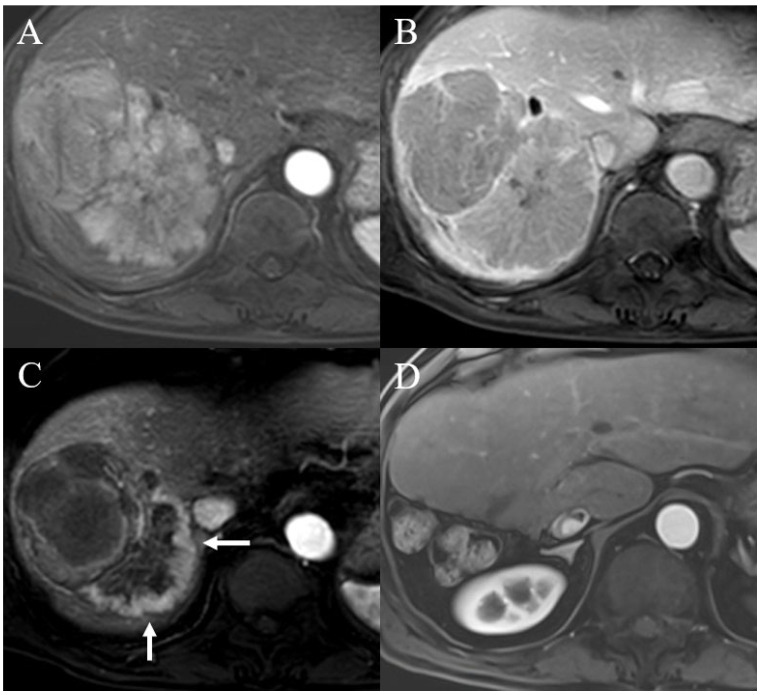
Case of a man who underwent transarterial radioembolization (TARE) for hepatocellular carcinoma (HCC). (**A**,**B**) Pre-procedure liver dynamic magnetic resonance imaging revealed a 12.8 cm HCC in segments VII and VIII. (**C**) On follow-up liver dynamic magnetic resonance imaging at 3 months after TARE, suspicious arterial enhancement (arrow) was noted at the inferior aspect of the treated lesion. Peripheral arterial enhancement was observed up to 10 months after TARE; thus, the patient subsequently underwent surgical resection, and viable HCC was confirmed on surgical pathology. (**D**) The patient was followed up until recently and has remained free of recurrence for 61 months after surgery.

Table 2 shows the univariable and multivariable regression analyses used to evaluate the predictors of tumor recurrence within 5 years after TARE. In the univariable regression analysis, the diameter of the largest tumor > 7 cm (OR = 16.949, 95% CI = 1.825–166.667, *p* = 0.013) and portal vein thrombosis (OR = 6.933, 95% CI = 0.762–63.047, *p* = 0.086) were related to tumor recurrence after TARE for HCC. However, in the multivariable analysis, only the diameter of the largest tumor > 7 cm was identified as a significantly related factor for tumor recurrence (OR = 21.277, 95% CI = 2.066–200.000, *p* = 0.010) (Figure 2 and Figure 3).

## 4. Discussion

This study evaluated preprocedural factors associated with long-term tumor control in patients with HCC treated with TARE. Among the 37 patients included, 24.3% (nine patients) achieved a CR, defined as no recurrence without additional treatment over 5 years (CR group). By contrast, 75.7% (28 patients) experienced recurrence and required additional treatment during the same period (the non-CR group). The largest tumor diameter was significantly larger in the non-CR group than in the CR group (9.8 cm vs. 5.9 cm, *p* = 0.006). Although portal vein thrombosis was more frequently observed in the non-CR group (46.4% vs. 11.1%), the difference was not statistically significant (*p* = 0.062). In multivariable analysis, tumor diameter > 7 cm was the only independent predictor of recurrence within 5 years after TARE.

These findings highlight the critical role of baseline tumor burden in predicting long-term oncological outcomes in patients with HCC treated with TARE. A large tumor size likely reflects not only the extent of the disease but also its biological aggressiveness, including increased intratumoral heterogeneity, hypoxic microenvironments, and resistance to radiation-based therapies. Tumors > 7 cm may exhibit suboptimal microsphere penetration and dose delivery because of central necrosis and irregular vascularity, ultimately reducing the efficacy of Y90 embolization. Moreover, large tumors may be associated with microscopic vascular invasion or satellite nodules that could not be detected on pre-procedure imaging, thereby contributing to high recurrence rates [15,25]. The fact that tumor size remained significant even in the multivariable analysis suggests its independent predictive value and the need to reconsider tumor size thresholds when selecting patients for TARE with curative intent. The result of our study is consistent with previous studies reporting that tumor size is associated with CR after TACE [26,27]. Although there were a few previous studies demonstrating the association between tumor size and CR following TARE [28], there are still only a limited number of studies investigating radiologic factors that can predict tumor response after TARE. Moreover, whereas earlier studies used 9 cm as the cutoff, our study adopted 7 cm as the cutoff. Therefore, our results may provide a more refined criterion regarding tumor size, and the accumulation of such data could contribute to the development of personalized treatment strategies.

Moreover, this study suggests the potential association between portal vein thrombosis and tumor response after TARE. Although portal vein thrombosis did not reach statistical significance in this study, its higher frequency in the non-CR group is consistent with the existing literature that reports that portal vein thrombosis might be a negative prognostic factor [29,30]. The presence of portal vein thrombosis is often indicative of advanced tumor biology and intrahepatic vascular invasion, which could predispose patients to rapid intrahepatic recurrence, even after initial tumor control. Moreover, even when portal vein thrombosis presents as bland thrombosis, it can still impair hepatic perfusion and limit the distribution of Y90 microspheres, potentially leading to subtherapeutic radiation doses in the tumor bed. Previous studies have identified portal vein thrombosis as a key determinant of both treatment response and survival in patients undergoing TACE [31]. Although our study did not demonstrate statistical significance, likely because of the limited sample size, the clinical relevance of portal vein thrombosis should not be underestimated. Similarly, although some previous studies have reported that low MELD score is associated with complete response after TARE [28,32], our study did not demonstrate a significant relationship. This discrepancy may be attributable to the small sample size of our cohort. Nonetheless, only limited evidence exists regarding the relationship between MELD score and tumor response after TARE; thus, additional studies are needed to clarify the underlying reasons for its potential association between MELD score and the probability of CR achievement.

The results of our study potentially offer supplementary guidance for clinical decision-making after TARE, especially when the assessment of early response is unclear. While TARE can be used as a palliative treatment or as a bridging therapy prior to liver transplantation, it can also be employed with curative intent in select cases, especially when surgical resection or liver transplantation is contraindicated [33,34]. However, evaluating the treatment response after TARE can be challenging. Postprocedural imaging often reveals equivocal peritumoral enhancement, which is difficult to distinguish from residual or recurrent tumor activity [10,11]. In clinical practice, physicians frequently initiate additional treatments, such as TACE or radiotherapy, when ambiguous enhancement is accompanied by rising tumor markers, especially in patients treated with curative intent. This common practice complicates the assessment of true long-term treatment outcomes, as patients who may have eventually demonstrated CR if monitored conservatively may undergo further premature therapy.

Because of these inherent limitations in post-TARE imaging interpretation, it is difficult to reliably assess treatment efficacy and predict which patients will achieve durable tumor control without additional interventions. Therefore, this study was designed to identify pretreatment factors that can predict 5-year CR following TARE. The ability to anticipate long-term CR would allow clinicians to adopt a more individualized management approach, potentially avoiding unnecessary additional treatment in patients with indeterminate imaging findings. For instance, in patients with tumors ≤ 7 cm and no evidence of portal vein thrombosis, even if peritumoral enhancement is observed in the early posttreatment period after TARE, a watchful waiting strategy might be a reasonable alternative to immediate retreatment.

Such an approach would support personalized treatment planning and follow-up strategies after TARE. Previous studies have attempted to predict treatment outcomes using radiomics-based imaging analyses; however, these analyses have several limitations, including a lack of standardization and reproducibility, insufficient external validation, and limited generalizability [35,36,37,38]. By contrast, this study focused on simple, easily identifiable imaging features, tumor size, and portal vein thrombosis, which can be readily assessed in routine clinical practice and may serve as reliable predictors of long-term tumor control after TARE.

This study has several limitations. One of the main limitations of this study was its retrospective design. Only patients who were followed for >5 years after TARE were included in the study. Thus, patients who experienced early recurrence or died within a shorter follow-up period were excluded. For patients lost to follow-up, the recurrence status could not be determined. This reflects an inherent limitation of the retrospective study design, and the selective case design introduces a potential selection bias. Prospective studies with standardized follow-up protocols are warranted to validate these findings. Second, the small sample size, particularly in the CR group (n = 9), due to the significant reduction in the study patients from the initial population. This may impact the interpretation of the results, including a wide confidence interval for the odds ratio of tumor size in multivariable analysis, raising concerns about potential statistical skewness, and limit the statistical power and generalizability of the findings. The sample size calculation revealed that at least 385 patients are required to have a confidence level of 95% [39]. While this study focused on identifying the key predictors of long-term CR, future studies with larger cohorts may evaluate additional variables and allow the development of predictive models to guide individualized post-TARE treatment planning. Third, our study cohort has a male predominance. Although no significant differences in sex distribution were observed between the CR and non-CR groups and univariable analysis did not demonstrate a significant association between sex and long-term tumor response, the under-representation of female patients limits the generalizability of our findings. Validation in larger and more balanced populations is warranted. Finally, radiologic features beyond tumor size and portal vein thrombosis, such as diffusion restriction characteristics or features based on radiomics analysis, were not evaluated but could yield additional predictive value. Future studies with larger multicenter cohorts and advanced imaging analyses are warranted to validate these results.

In conclusion, our study demonstrates that tumor diameter > 7 cm is a significant predictor of tumor recurrence within 5 years after TARE for HCC. Conversely, in patients with tumors ≤ 7 cm, equivocal hyperenhancement observed on early follow-up imaging may not necessitate immediate additional treatment. These findings support a more individualized approach to post-TARE management, potentially allowing clinicians to avoid overtreatment and adopt a watchful waiting strategy for selected patients.

## Figures and Tables

**Table 1:** Baseline characteristics of the study population.

Characteristic	CR Group	Non-CR Group	*p* Value	Total
No. of patients	9	28		37
Age (years)	65 (52–69)	64 (54–74)	0.726	64 (53–72)
Sex (men), n (%)	7 (77.8)	23 (82.1)	0.556	30 (81.1)
Cirrhosis, n (%)	5 (55.6)	15 (53.6)	0.612	20 (54.1)
Child–Pugh class, n (%)			0.578	
A	8 (88.9)	26 (92.9)		34 (91.9)
B	1 (11.1)	2 (7.1)		3 (8.1)
MELD score	7.4 (6.0–8.0)	7.5 (6.0–8.0)	0.889	7.5 (6.0–8.0)
Tumor characteristics				
Diameter of largest tumor (cm)	5.9 (4.8–6.8)	9.8 (6.8–12.0)	0.006	7.6 (6.2–11.6)
Diameter of largest tumor, n (%)			0.004	
≤7 cm	8 (88.9)	9 (32.1)		17 (45.9)
>7 cm	1 (11.1)	19 (67.9)		20 (54.1)
Tumor epicenter, n (%)			0.444	
Right	8 (88.9)	22 (78.6)		30 (81.1)
Left	1 (11.1)	6 (21.4)		7 (18.9)
Number of main tumors	1 (1–1)	1 (1–2)	0.256	2 (1–2)
Presence of satellite nodule, n (%)	1 (11.1)	6 (21.4)	0.444	7 (18.9)
Tumor enhancement pattern, n (%)			0.432	
Typical enhancement	8 (88.9)	27 (96.4)		35 (94.6)
Atypical enhancement	1 (11.1)	1 (3.6)		2 (5.4)
Tumor margin, n (%)			0.543	
Smooth	8 (88.9)	23 (82.1)		31 (83.8)
Irregular	1 (11.1)	5 (17.9)		6 (16.2)
Portal vein thrombosis, n (%)	1 (11.1)	13 (46.4)	0.062	14 (37.8)
Hepatic vein invasion, n (%)	1 (11.1)	1 (3.6)	0.432	2 (5.4)
Ascites			0.471	
None	5 (55.6)	10 (35.7)		15 (40.5)
Small amount	4 (44.4)	16 (57.1)		20 (54.1)
Moderate to large amount	0 (0.0)	2 (7.1)		2 (5.4)
Laboratory data				
Alpha-fetoprotein (ng/mL)	2.7 (2.3–5.2)	3.1 (2.0–6.2)	0.419	3.0 (2.2–5.5)
PIVKA-II (mAU/mL)	153.0 (22.0–14,430.0)	316.5 (103.8–3766.0)	0.232	302.0 (77.0–3677.0)
Total bilirubin (mg/dL)	0.9 (0.7–1.3)	0.8 (0.5–1.0)	0.915	0.8 (0.6–1.0)
Albumin (g/dL)	3.7 (3.3–4.1)	3.7 (3.3–4.0)	0.654	3.7 (3.3–4.0)
AST (IU/L)	49.0 (35.0–54.0)	55.0 (32.3–81.5)	0.319	52.0 (32.5–77.0)
ALT (IU/L)	24.0 (19.5–42.5)	32.5 (19.3–40.8)	0.336	31.0 (19.5–40.5)
Platelet (1000/μL)	221.0 (119.5–256.0)	195.5 (169.5–274.0)	0.345	202.0 (157.0–272.0)
PT-INR	1.0 (0.9–1.1)	1.0 (1.0–1.1)	0.664	1.0 (1.0–1.1)
Interval periods (days)	20 (16–22)	16 (13–21)	0.102	17 (13–21)
Lung shunt (%)	5.7 (4.2–9.8)	4.9 (3.2–7.6)	0.759	5.3 (3.4–7.9)

Continuous variables are expressed as medians with interquartile ranges, and categorical variables are expressed as absolute numbers with percentages. Continuous variables were compared using the Mann–Whitney U-test or Kruskal–Wallis test, whereas categorical variables were compared using the chi-squared test or Fisher’s exact test. CR, complete response; MELD, model for end-stage liver disease; PIVKA, prothrombin induced by vitamin K absence or antagonist; AST, aspartate aminotransferase; ALT, alanine aminotransferase; PT-INR, prothrombin time–international normalized ratio. Interval period = time interval from the date of pre-procedure magnetic resonance imaging to the date of the transarterial radioembolization procedure.

**Table 2:** Univariable and multivariable analysis for predictors of 5-year non-CR after TARE for HCC.

Variables	Univariable Analysis			Multivariable Analysis		
	OR	95% CI	*p* Value	OR	95% CI	*p* Value
Age	0.874	0.780–0.979	0.556			
MELD score	0.956	0.856–1.055	0.757			
Diameter of largest tumor						
≤7 cm	Reference					
>7 cm	16.949	1.825–166.667	0.013	21.277	2.066–200.000	0.010
Number of main tumors	4.293	0.503–36.673	0.183			
Presence of satellite nodule	0.458	0.048–4.420	0.500			
Portal vein thrombosis	6.933	0.762–63.047	0.086	9.779	0.881–108.595	0.063
Hepatic vein invasion	3.375	0.189–60.238	0.408			
Alpha-fetoprotein (ng/mL)	1.051	0.871–1.267	0.605			
PIVKA-II	2.852	0.973–7.330	0.244			
Interval periods	0.862	0.719–1.033	0.108			

CR, complete response; TARE, transarterial radioembolization; HCC, hepatocellular carcinoma; OR, odds ratio; CI, confidence interval; MELD, model for end-stage liver disease; PIVKA, prothrombin induced by vitamin K absence or antagonist. Interval period = time interval from the date of preprocedure magnetic resonance imagining to the date of the TARE procedure.

## Data Availability

The data that support the findings of this study are available from the corresponding author, [Kim DK], upon reasonable request.

## Supplementary Materials

The following supporting information can be downloaded at: https://www.mdpi.com/article/10.3390/diagnostics15182297/s1, Table S1: Parameters of contrast-enhanced liver dynamic MRI.
