# Supplementary material for: Assessment of Preprocedural Factors Associated with 5-Year Complete Response After Transarterial Radioembolization in Patients with Hepatocellular Carcinoma
Source: Diagnostics (Basel). 2025 Sep 10;15(18):2297. doi: 10.3390/diagnostics15182297 (PMC12468652; doi:10.3390/diagnostics15182297)
Supplement: Supplementary file 1 [file diagnostics-15-02297-s001.zip › diagnostics-3781634-supplementary.pdf]

Supplementary Table S1. Parameters of contrast-enhanced liver dynamic MRI

Sequence	Scanner	Matrix Size	ST (mm)	Gap (mm)	TR (msec)	TE (msec)	FA (°)
Dual-echo T1-weighted GRE	SIGNA Premier	280 × 280	5	2.5	4.78	1.39/2.82	12
	Ingenia Elition X	320 × 290	3	3	3.62	1.19/2.37	10
T1-weighted 3D GRE with dynamic enhancement	SIGNA Premier	300 × 280	3	0	3.39	1.54	12
	Ingenia Elition X	256 × 283	3	0	3.72	1.47	10
T2-weighted turbo spin echo	SIGNA Premier	300 × 300	5	6	680	90	90
	Ingenia Elition X	324 × 290	5	6	536	80	90
DWI	SIGNA Premier	320 ×320	5	6	717	82	90
	Ingenia Elition X	324 × 290	5	6	680	80	90

Abbreviations: 3D, three-dimensional, ST, slice thickness; TR, repetition time; TE, echo time; FA, flip angle; GRE, gradient-recalled echo; DWI, diffusion-weighted imaging
